# Acaricidal effect of major compounds to control *Rhipicephalus microplus* (Canestrini, 1887) in dairy cows and possible alternatives for reversing multidrug resistance

**DOI:** 10.1590/S1984-29612022028

**Published:** 2022-05-27

**Authors:** Carla Juliana Ribeiro Dolenga, Alan dos Anjos, Victor Hugo Granza Barbosa, Ursula Yaeko Yoshitani, Prisciane Leme da Silva Piuci Castilho, Vanessa Issuzu Miyakawa, Marcelo Beltrão Molento

**Affiliations:** 1 Laboratório de Parasitologia Clínica Veterinária, Departamento de Medicina Veterinária, Universidade Federal do Paraná – UFPR, Curitiba, PR, Brasil; 2 Médica Veterinária autônoma, Santana do Itararé, PR, Brasil

**Keywords:** Adult immersion test, ectoparasite resistance, cattle tick, ruminants, sustainable farming, Teste de imersão de adultos, ectoparasitas resistentes, carrapato bovino, ruminantes, agricultura sustentável

## Abstract

Tick control represent a great challenge to animal health. The objective of this study was to evaluate the efficacy of acaricidal compounds against *Rhipicephalus microplus* from dairy cattle systems in Paraná State, Brazil. Six farms (PR1-PR6) were selected, where anti-tick products were applied at fixed intervals. Two other farms that adopted alternative protocols; target selective treatment (PRS), and individual-based agroecological protocol (PRA) were also included in the trial. Fully engorged *R. microplus* were collected for the *in vitro* adult immersion test (AIT), and the egg hatch test (EHT) in all eight populations. The larval packet test (LPT) was used on PR6 and PRA tick populations. The treatment groups were composed of G1: dichlorvos 45% plus cypermethrin 5%, G2: deltamethrin 2.5%, G3: cypermethrin 15%, chlorpyriphos 25%, plus citronellal 1%, and G4: amitraz 12.5%. The efficacy at PR1 to PR6 revealed that G3 and G4 achieved moderate to high efficacy, from 75.0 to 100.0% and 73 to 98%, respectively. In the LPT, the efficacy at PR6 was 76.0, 67.0, 93.0 and 30.6%, while PRA presented 100.0, 100.0, 100.0, and 54.0%, for G1, G2, G3 and G4, respectively. Sustainable parasite control strategies are discussed.

## Resumo

O controle do carrapato representa um grande desafio na saúde animal. O objetivo deste trabalho foi avaliar a eficácia de compostos acaricidas contra *Rhipicephalus microplus* em sistemas de pecuária leiteira no Paraná, Brasil. Foram selecionadas seis fazendas (PR1-PR6), onde são aplicados produtos carrapaticidas regularmente. Duas outras fazendas que adotaram protocolos alternativos, PRS (tratamento seletivo) e PRA (controle agroecológico individual), também foram incluídas nos testes. *R. microplus* ingurgitadas foram coletados para testes *in vitro* de imersão em adultos (TIA) e teste de eclosão de ovos (TEO) em todas as oito populações. O teste de envelope de larvas (TEL) foi utilizado nas populações PR6 e PRA. Os grupos de tratamento foram compostos por G1: diclorvos 45% mais cipermetrina 5%, G2: deltametrina 2,5%, G3: cipermetrina 15%, clorpirifos 25% e mais citronelal 1% e G4: amitraz 12,5%. A eficácia em PR1 a PR6 revelou que G3 e G4 obtiveram eficácia de moderada a alta, variando de 75,0% a 100,0% e 73% a 98%, respectivamente. No TEL, a eficácia em PR6 foi de 76,0%, 67,0%, 93,0% e 30,6%, enquanto em PRA apresentou 100,0%, 100,0%, 100,0% e 54,0%, para G1, G2, G3 e G4, respectivamente. Estratégias sustentáveis de controle são discutidas neste documento.

## Introduction

Tick infestation is a critical parasite problem and is considered an obstacle to animal welfare and their ability to transmit diseases (Moumouni et al., 2018). The cattle tick, *Rhipicephalus microplus* (Canestrini, 1887, Acari, Ixodidae) is widely distributed in South and Central America causing large economic losses (Barros-Battesti et al., 2006). The parasite is commonly found in Brazil, causing an estimated impact of US$ 3.2 billion/year (Grisi et al., 2014). Other problems related to its presence are opportunistic bacterial infections, weight loss, and myiasis (Garcia et al., 2019).

The most common method of *R. microplus* control is the use of commercial acaricides. Unfortunately, the excessive use of these agents has imposed the selection of resistant parasites, also resulting in high residue levels in milk and meat (Souza et al., 2017). Camillo et al. (2008), showed a lack of efficacy in more than 90% of the farms in Rio Grande do Sul, and this was also observed by Mendes et al. (2007), in São Paulo, Brazil. As an option for maintaining efficient tick control and to counteract parasite resistance, farmers and practitioners are indicating products in combination with a different mode of action (i.e. pyrethroids and organophosphates), and in higher concentrations (M.B. Molento, personal observation). Barré et al. (2008), have demonstrated the synergism effect of amitraz *in vitro* when it was used in combination with deltamethrin against larvae of *R. microplus*, enhancing deltamethrin toxicity.

According to a more holistic approach, Dumont et al. (2013), proposed the adoption of management practices that could improve animal health, decreasing negative inputs to the system. The authors suggested that one should strengthen diversity to increase animal and farm resilience, while preserving biological diversity in agroecosystems. Moreover, biological, biodynamic, and agroecological farming using sustainable protocols may aid to reduce the dependency of chemical use, and promote biodiversity and resilient environments assisting the host-parasite relationship (Lampkin et al., 2015). These principles follow Molento (2009) where welfare is an important issue that must be considered if we agree to maintain animals with tolerable levels of parasitism. These animal husbandry systems are also quite beneficial, as partial selective treatment would maintain tick populations in refugia by preserving susceptible parasites unexposed to chemical compounds (Molento et al., 2013; Rodriguez-Vivas et al., 2018; Sissay et al., 2006). The objective of the present study was to determine the efficacy of major acaricides against different stages of *R. microplus* from dairy cattle. We also compare the study data to an agroecological and a selective system, suggesting sustainable tick control alternatives.

## Material and Methods

### Farm description and management

**Figure 1:**
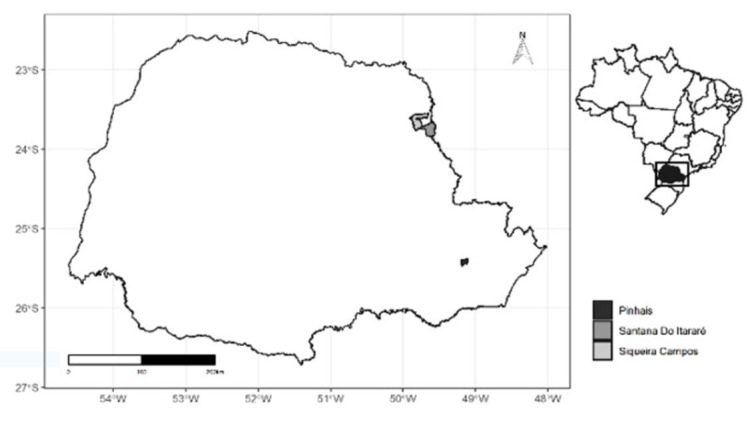
Location of the state of Paraná in Brazil and the respective municipalities of Pinhais, Siqueira Campos, and Santana do Itararé where ticks were collected.

Dairy farms (PR1 to PR6) had a range of 14 to 105 animals with an average production of 12 to 28 kg/milk/day. Tick control was based on intensive treatment with short intervals (15 to 40 days) of multiple products (Table 1). Fully engorged female of *R. microplus* ticks (approx. 250/farm) were collected from the six dairy cattle farms, and one from selective (PRS), and one agroecological (PRA) farm from Paraná State (PR), south of Brazil. The PRS used target selective treatment based on a visual inspection with a limit of 20 ticks on one side of the animal (Molento et al., 2013), and the PRA used biocontrol (local organic compost) and phytotherapy (*Cymbopogon citratus*) locally prepared for tick control. PRA used to be a typical research farm until 2004 when animals were regularly treated with acaricides (cypermethrin and amitraz). Animals from PRA were not treated with any commercial acaricide since – therefore, the ticks that were collected for the test had no selection or exposure to acaricides for the last 15 years. PR1 to PR5 were located in the city of Santana of Itararé, PR6 was located in the city of Siqueira Campos, and PRS and PRA were located in Pinhais, PR (Figure 1).

**Table 1:** Description of each farm, including the number of animals (N), type of exploration, average of milk production (kg/day), management system, previous acaricide* use, and the treatment intervals in days.

Farm	N	Type	Milk Prod.	System	Acaricide*	Freq/days
PRA	25	Dairy	300	Agroecol.	Phytotherapy	30
PR1	45	Dairy	1100	Intensive	D/F/FL+A	30
PR2	68	Dairy	1520	Intensive	AM/E+CH+AL/FL/D	15
PR3	70	Dairy	1700	Intensive	CY+CH+PB/FL/FL+F/I	30
PR4	18	Dairy	380	Intensive	D/ CY-CH+PB	40
PR5	14	Dairy	300	Intensive	CY+CH+CT/CY+CH+FL+PB	30
PR6	105	Dairy	2940	Intensive	AM/CY/D/F	30
PRS	48	Beef	---	Integrated	CY, I	Selective

CY: cypermethrin; CH: chlorpyriphos; PB: piperonyl butoxide; CT citronellal; D: doramectin; E: ethion; AL: alfacypermethrin; F: fipronil; FL: fluazuron; A: abamectin; I: ivermectin.

* AM: amitraz.

Table 1. Description of each farm, including the number of animals (N), type of exploration, average milk production (kg/day), management system, previous acaricide* use, and the treatment intervals in days.

### Treatment groups

The ticks were manually collected from naturally infected animals and were divided into five treatment groups, G1: a combination of dichlorvos 45% and cypermethrin 5% (Alatox, Zoetis Animal Health), G2: deltamethrin 2.5% (Butox CE 25, MSD Saúde Animal), G3: a combination of cypermethrin 15%, chlorpyriphos 25%, and citronellal 1% (Colosso, Ouro Fino Saúde Animal), and G4: amitraz 12.5% (Triatox, MSD Saúde Animal). A control group (NC) using distilled water was added to every test. Each treatment and the control group were homogeneously formed (n=10) with similar tick size and weight. The experimental groups of ticks were tested in triplicates. Products with different mechanisms of action and compound combinations were chosen for the text, following the manufacturers’ recommendation.

Figure 1. Localities in the Paraná State, Brazil and the respective municipalities of Pinhais, Siqueira Campos, and Santana do Itararé where ticks were collected.

### Adult Immersion Test (AIT)

The AIT is based on the evaluation of two different life stages to determine the efficacy of each product (Drummond et al., 1973). For this test, ticks were submitted to an immersion step containing 10 ml of the product concentrations based on the manufacturer’ recommendation for 5 min, including a gentle agitation every 30 seconds. Later, the ticks were removed from the solution, briefly washed under distilled water, dried in filter paper, and placed in *Petri* dishes. The ticks were kept for 14 days in an incubator at 27º C with 80% relative humidity for complete tick oviposition. The number of viable, not-viable, incomplete, or no oviposition was determined at this time.

After this, 0.3 g of viable eggs from each group were transferred to 15 ml Falcon tubes, covered with hydrophobic cotton, and returned to the incubator under the same conditions. The ticks were kept for 28 days to measure the percentage of egg hatching. The visual reading of the hatchability was done by comparing the number of visible larvae and shells with unhatched eggs, using a 5% interval. From this data, Equations 1 and 2 were used to calculate the reproductive index (RI) and the overall efficacy of the product (E%) (Drummond et al., 1973):

Equation 1:


$$RI\;=\;\left(total\;weight\;of\;eggs\;\left(g\right)\;x\;hatching\;\left(\%\right)\;x\;20.000\right)\;/Tick\;weight\;\left(g\right)$$ (1)

Equation 2:


$$E\%\;=\;\left(control\;group\;RI\;–\;treated\;group\;RI\right)\;/\;control\;group\;x\;100$$ (2)

### Larval Packet Test (LPT)

The original technique was described by Stone & Haydock (1962) to test the effect of acaricides on the free-living tick larvae. This test is efficient and low-cost. The LPT was used in one suppressive (PR6) and the agroecological (PRA) tick populations.

One hundred fresh tick larvae (7 days of hatching) were obtained and placed in filter paper that was soaked with 100 µl of each of the above-listed products (G1, G2, G3, and G4). Dilutions were based on the manufacturer’s recommendation. The filter papers were then folded similar to envelopes and were sealed with metal staples to prevent the larvae from escaping. The envelopes were incubated at 27ºC with 70% humidity for 24 h. The test was interpreted by identifying live and dead larvae with the aid of a vacuum pump. Inactive parasite larvae were considered dead after 5 seconds. The entire protocol was performed in triplicate, and Equation 3 was used to calculate the group efficacy:

Equation 3:


$$Mortality\;\left(\%\right)\;=\;\left(total\;number\;of\;dead\;larvae\;/\;total\;number\;of\;larvae\right)\;x\;100$$ (3)

### Statistical analysis

The analysis of variance (ANOVA) was run followed by the Tukey test. The assumptions, homogeneity of variances, and normality were evaluated by the Barlett test and the Shapiro-Wilk method. The analysis of residues, normality and the coefficient of determination (R^2^) were performed to establish the best model. The tests were carried out with the aid of the “agricolae” package with the highest value being the referential (letter “a”) (Mendiburu, 2017). The data manipulation was performed using the “tidyverse” package (Wickham, 2017). The results obtained for the AIT for PR1 to PR6, PRS, and PRA were compared using the R Core Team (2019) software.

## Results

### Adult immersion test

Drug performance was significantly different between farms and according to the system (Table 2), having poor to highly toxic effects. PRA presented close to 100% efficacy of all products. Although PR1 and PR2 showed similar data, from 20 to 77% and from 23 to 73%, respectively, there was no major egg-laying reduction for any compound. The PR3 and PRS presented the lowest efficacy rates when looking at all tested products (from 7 to 53%), obtaining poor toxicity against adult ticks. The AIT revealed that G1 (worse performance) and G4 (best performance) drugs, did not demonstrate significant statistical variation (*P* > 0.05) between them, with consistently low or moderate to high efficacy. On the other hand, G2 and G3 showed significant differences (*P* < 0.05) among locations (from 17 to 100%), possibly reflecting an active on-farm population selection process.

**Table 2:** Percentage (%) and standard deviation (+/-) of acaricide compounds (G1, G2, G3, G4) using the adult immersion test (AIT), the egg hatch test (EHT) and the overall efficacy (OE) for *Rhipicephalus microplus* from an agroecological (PRA), selective (PRS), and intensive (PR1 to PR6) dairy farms in Paraná, Brazil.

Tests	Farms	Products							
		G1		G2		G3		G4	
AIT	PRA	37.0 (±40.0)	bcA	77.0 (±6.0)	abA	100.0 (±0.0)	aA	90.0 (±0.0)	aA
	PR1	20.0 (±20.0)	bcA	30.0 (±10.0)	bcAB	53.0 (±12.0)	abBC	77.0 (±21.0)	aA
	PR2	23.0 (±15.0)	bA	30.0 (±17.0)	bAB	70.0 (±10.0)	aB	73.0 (±6.0)	aA
	PR3	7.0 (±6.0)	aA	27.0 (±25.0)	aAB	47.0 (±12.0)	aBC	53.0 (±35.0)	aA
	PR4	3.0 (±6.0)	bA	13.0 (±12.0)	bB	53.0 (±12.0)	aBC	73.0 (±15.0)	aA
	PR5	10.0 (±17.0)	bA	27.0 (±25.0)	abAB	53.0 (±6.0)	abBC	60.0 (±17.0)	aA
	PR6	20.0 (±10.0)	bA	20.0 (±26.0)	bB	33.0 (±12.0)	abC	67.0 (±21.0)	aA
	PRS	17.0 (±19.0)	aA	17.0 (±14.0)	aB	25.0 (±12.0)	aC	33.0 (±26.0)	aA
EHT	PRA	13.0 (±15.0)	bB	13.0 (±14.0)	bB	0.0 (±0.0)	bA	0.0 (±0.0)	bA
	PR1	90.0 (±0.0)	aA	60.0 (±36.0)	abAB	33.0 (±40.0)	abA	13.0 (±12.0)	bA
	PR2	73.0 (±6.0)	aA	80.0 (±17.0)	aA	27.0 (±38.0)	aA	40.0 (±30.0)	aA
	PR3	80.0 (±0.0)	aA	57.0 (±15.0)	aAB	50.0 (±26.0)	aA	63.0 (±46.0)	aA
	PR4	10.0 (±10.0)	abB	10.0 (±10.0)	abB	0.0 (±0.0)	bA	10.0 (±10.0)	abA
	PR5	60.0 (±17.0)	abAB	13.0 (±15.0)	bB	23.0 (±15.0)	abA	33.0 (±29.0)	abA
	PR6	80.0 (±9.0)	abA	48.0 (±24.0)	bcAB	13.0 (±8.0)	dA	18.0 (±6.0)	cdA
	PRS	60.0 (±44.0)	aAB	53.0 (±29.0)	aAB	37.0 (±47.0)	aA	33.0 (±40.0)	aA
OE	PRA	86.0 (±16.0)	aAB	93.0 (±9.0)	aA	100.0 (±0.0)	aA	100.0 (±0.0)	aA
	PR1	29.0 (±26.0)	bcABC	58.0 (±30.0)	abAB	87.0 (±17.0)	aA	98.0 (±3.0)	aA
	PR2	43.0 (±19.0)	bcABC	40.0 (±26.0)	bAB	89.0 (±18.0)	aA	91.0 (±8.0)	aA
	PR3	25.0 (±1.0)	bcBC	56.0 (±18.0)	abAB	75.0 (±16.0)	aA	73.0 (±33.0)	abA
	PR4	90.0 (±9.0)	aA	88.0 (±13.0)	aAB	100.0 (±0.0)	aA	96.0 (±4.0)	aA
	PR5	38.0 (±7.0)	bABC	87.0 (±16.0)	aAB	84.0 (±13.0)	aA	80.0 (±17.0)	aA
	PR6	6.0 (±6.0)	bcC	42.0 (±28.0)	bAB	84.0 (±9.0)	aA	81.0 (±11.0)	aA
	PRS	28.0 (±49.0)	aBC	23.0 (±35.0)	aB	58.0 (±52.0)	aA	60.0 (±52.0)	aA

Obs. Lower case letters represent the comparison of the tested groups within the farm (line), while upper case letters demonstrate the variations among farms for each isolated drug (column). Different letters represent statistically significant differences (*P* < 0.05). The statistical analysis is not between tests.

The egg hatch demonstrated that most of the drugs were significantly different between and within farms (Table 2). Overall, the PRA presented the highest efficacy with the lowest egg hatch rates. PRA was similar (*P* > 0.05) to PR1, PR4, PR5, and PR6 for all products. The previous farms used at least 30-day treatment intervals. The efficacy on PR2, PR3, and PRS was similar (*P* > 0.05), presenting high egg hatch and the lowest efficacy rates.

The efficacy obtained for G3 and G4 did not demonstrate any statistical difference (*P* > 0.05). On the other hand, G1 and G2 were significantly different (*P* < 0.05) among the locations, showing a wide range of egg hatch rates (10 to 90%). Similar to the AIT, the drugs that selected the least had no egg hatch difference – describing the beginning of a selection process with no phenotypic variation (G3 and G4).

Although in the PRA we found the highest efficacies for all products, there was no statistical difference (*P* > 0.05) between G3 and G4, and of some farms for G1 and G2. The PR3, PR6, and PRS presented the lowest efficacy rates for the tested chemical groups (Table 2). Cypermethrin and chlorpyriphos/G3 and amitraz/G4 had a low to very high efficacy (from 58 to 100%). The efficiency obtained from G3 and G4 did not demonstrate a statistical difference between farms (best performance). Deltamethrin/G2 used alone showed poor to adequate efficacy (23 to 93%). The combination of cypermethrin and dichlorvos/G1 had the poorest performance, reaching an adequate efficacy at PR4 (06 to 90%) (Table 2).

### Larval packet test

**Figure 2:**
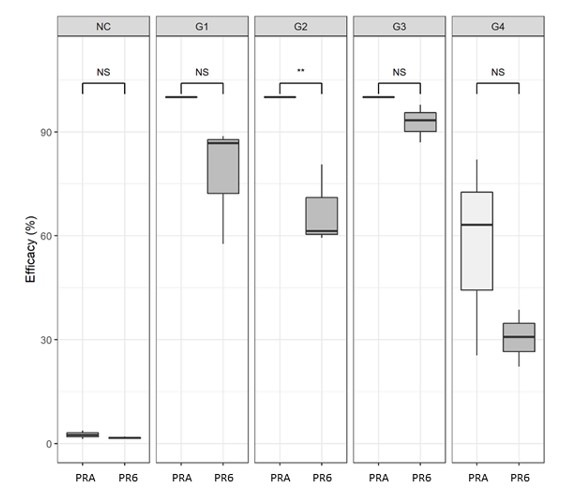
Efficacy (%) of acaricide products (G1, G2, G3, G4) including the control group (NC) against larvae of *Rhipicephalus microplus* from an agroecological (PRA) and an intensive (PR6) dairy farm in Paraná, Brazil. NS: not significantly different. ** = *P* < 0.01.

Although the PRA system showed constant superior efficacies when compared to PR6, the data revealed a statistical difference (*P* < 0.05) only for deltamethrin/G2 (Figure 2). G3 presented the best larval mortality (> 90%) on both farms. G4 had the lowest mortality in both farms as well. G4 was also statistically different (*P* < 0.05) from all the other treatments in each farm (data not shown), suggesting the maintenance of a strong resistance phenotypic background to amitraz even at the PRA.

Table 2. Percentage (%) and standard deviation (+/-) of acaricide compounds (G1, G2, G3, G4) using the adult immersion test (AIT), the egg hatch test (EHT), and the overall efficacy (OE) for *Rhipicephalus microplus* from an agroecological (PRA), selective (PRS), and intensive (PR1 to PR6) dairy farms in Paraná, Brazil.

Figure 2. Efficacy (%) of acaricide products (G1, G2, G3, G4) including the control group (NC) against larvae of *Rhipicephalus microplus* from an agroecological (PRA) and an intensive (PR6) dairy farm in Paraná, Brazil.

## Discussion

### Acaricide data

The data obtained in this study showed a wide variation in the efficacy of the different acaricides, reflecting farm management and drug pressure. Our data indicate that amitraz was the most toxic drug against adult ticks. Similar variations were also reported by Higa et al. (2020), using the AIT and the LPT in *R. microplus*. The authors reported an efficacy of 98.8 and 100% for dichlorvos alone and in combination with chlorpyriphos. In our study, the combination of cypermethrin, chlorpyriphos and, citronellal (G3) had a low effect on egg hatch. In the LPT, on the other hand, G4 achieved the lowest mortality, reinforcing the previous statement that amitraz would be more effective against adult ticks, affecting the production of eggs. Higa et al. (2020) suggested that resistance may increase in *R. microplus* because the ticks would remain on the host for a longer period, being extensively exposed to treatments, suffering a higher selection pressure. Klafke et al. (2017), also identified a low level of mortality for amitraz (LPT) in two multidrug resistant tick populations in southern Brazil.

Overall, G1 obtained the lowest efficacy against adult ticks but had moderate mortality against larvae. G4, a formamidine, was the most effective in the AIT and, together with G3, a combination of an organophosphate and a pyrethroid, in the EHT. On the other hand, G4 differed significantly from all the other products (LPT), suggesting a different level of resistance to formamidines, organophosphates, and pyrethroids between the larval populations. G1 and G3 have both cypermethrin in their composition, differing in their concentrations (5 and 15%, respectively), and are also composed of organophosphates; dichlorvos 45% and chlorpyriphos 25%, respectively.

Our data shows that G1 had low values in the AIT and on the overall efficacy. G3 demonstrated the opposite in the EHT, and both drugs remained statistically similar when using the LPT. This might be due to the higher concentration of cypermethrin in G3, although these compounds have not been assessed alone. The different combinations of organophosphates, both with cypermethrin, might have influenced the efficacies of G1 and G3. Klafke et al. (2017) tested organophosphates and pyrethroids alone and obtained lower LC_50_ values for cypermethrin (2.98 and 1.32), than for chlorpyriphos (4.98 and 6.89). Similarly, Reck et al. (2014), using a resistant *R. microplus* population, found a similar LC_50_ value when using a lower concentration of cypermethrin (0.79), then for chlorpyriphos (1.45).

Although G1 presents dichlorvos in a higher concentration than chlorpyriphos in G3, these organophosphates may behave differently. G2 consisted of deltamethrin at 2.5%, and it presented the highest mortality in the LPT. G2 had higher efficacy in the AIT and EHT when compared to G1. Considering that both groups have pyrethroids in their composition and that G2 has a lower concentration, deltamethrin may be more effective than cypermethrin with an organophosphate of older generations, such as chlorpyriphos and dichlorvos.

### Farm data: differences, similarities, and possible solutions

Tick prevalence is greatly influenced by local climate and host management (Mastropaolo et al., 2017). PR1 to PR6 adopted a suppressive control probably because of their location in the north of Parana, where ticks are exposed to harsh conditions of dry winters and very hot summers (> 28^o^ C). PRA and PRS, in contrast, are relatively close to one another, and ticks live in cold (> 14^o^ C) and dry winters with short hot (> 22^o^ C) and humid summers. PR1 to PR5 are located in the same geographical region coexisting under related climate conditions, which we expected that tick populations would behave similarly.

The differences in efficacy obtained from some of these places are probably due to their unique acaricide frequency (15 to 45-day intervals). The farmer at PR6 also adopted the intensive system, although it differs from the others since it possibly faces phenotypically different tick populations. This fact would explain the low overall drug efficacies compared to the other intensive protocols. PRA, the only agroecological farm, presented the highest efficacy rates. The data is probably a consequence of the low drug selection as the population has not been exposed to conventional acaricides for over 15 years. This fact would account for more than 40 tick-generations (Maciel et al., 2015). PRS showed the lowest efficacy indicating the previous history of the intensive use of acaricides.

Both PRA and PRS were exposed to the same climatic conditions and vegetation, and the animals had similar tick challenges. Thus, one may assume that the contrasts observed in the tests were essentially, but not only, due to the treatment regime of these farms. In this scenario, one could expect the reversion of resistance, since the newest tick generations would not be selected by the chemicals. Maciel et al. (2015) analyzed *R. microplus* populations that had not been treated with amidines for 10 years. In the beginning, the population became resistant to amitraz after short-treatment intervals (every 28 days) between 2002 and 2004. The AIT was performed to assess the sensitivity of amitraz in 2005 and 2015, indicating a decrease in the efficacy from 70.0 to 62.0%, respectively. The data reinforces the importance of knowing the population background to establish an efficient tick control program (Kumar et al., 2020).

The suppressive use of chemicals in PR1 to PR6 and PRS has influenced the effectiveness of the compounds. Notably, the ticks developed multidrug resistance to acaricides, either by drug exposure in sequential treatments or products that offer drug combinations in their formulations. Moreover, greater efficacy was observed for all the products tested against the PRA strain. Only G3/cypermethrin, chlorpyriphos, and citronellal would be the product of choice for these systems. However, G3 did not demonstrate an efficacy above 95% in all farms (PR1 to PR6 and PRS). For PRA, both G3 and G4 treatments were highly effective against engorged females and eggs. However, G4 did not achieve mortality above 95% in the LPT for PR6.

Similar to PR6, Neto & Toledo-Pinto (2007) collected ticks from dairy farms and performed the AIT. The authors found efficacies of 98% for the combination of cypermethrin, chlorpyriphos, and citronellal, 20% for amitraz, 8% for the combination of dichlorvos and cypermethrin, and 0% for deltamethrin. Camillo et al. (2008) has analyzed the efficacy of cypermethrin, chlorpyriphos with citronellal, and amitraz using the AIT in 42 farms. The authors observed efficacies above 95% for the triple combination in 25 locations and only in 6 for amitraz. Pereira (2006), analyzed the efficacy of some products in dairy cattle and found efficacies of 25.4% for deltamethrin, 47.2% for amitraz, and 89% for the combination of cypermethrin, chlorpyrifos, and citronellal. The data for deltamethrin and amitraz were lower than those obtained in our study.

PRA showed very high efficacies to the most common drugs compared to the other tick populations. PR4, which used the 40-day drug interval, presented the second-best *in vitro* results. These data corroborate with Abbas et al. (2014), who concluded that the frequent use of the same acaricide for a long time is the most significant management factor in selecting for tick resistance. In this sense, integrated approaches to tick control may prove very effective (Muhammad et al., 2008). Even though our data need to be taken with caution, it suggests that agroecological (no chemical) and long treatment intervals are sustainable parasite control alternatives that can recover acaricide efficacy and reverse phenotypic multidrug resistance conditions.

Amaral et al. (2011), applied questionnaires to dairy farmers about their perspectives on strategic tick control and found that 92.4% that used this method noticed a decrease in tick numbers. They also found that producers were motivated to follow this strategy because it would reduce costs (46.6%), widen treatment intervals (44.2%), and require less labor (22.7%). Biocontrol is also an option, and Webster et al. (2015) assessed the use of *Metarhizium anisopliae* alone or in association with cypermethrin and chlorpyriphos against resistant *R. microplus* strains in field conditions. The biocontrol alone, the acaricides alone, and the combination reached 56.3, 71.1, and 97.9% of efficacy, showing an additive result. Molento et al. (2013) reported the successful use of a partial selective treatment strategy in a large-scale study. The authors have identified the most infested animals in beef cattle farms and demonstrated that treatments were never over 52% of the total herd.

The strict use of chemical control in the intensive systems was the differential factor for the selection of multidrug resistance in the PR1 to PR6 and PRS farms. The absolute restriction of acaricide in PRA indicates that drugs could have a much longer duration if products were used judiciously together with other local strategies (Graf et al., 2004; Molento et al., 2013). PRA data also showed that the strict reduction of drug use allowed the reversion of efficacy through natural phenotypic populational dilution to most of the tested products. This important strategy reflects the possible return of efficacy using the refugia concept (Sissay et al., 2006).

Drug resistance is a major concern among dairy cattle farmers. Thus, we encourage tick control to be done using individual evaluations, based on transient threshold abundance (Schafaschek et al., 2021) aiming at reducing the overall selection pressure, and preserving parasites in refugia. The differences between agroecological and intensive systems related to the development of drug resistance need to be further studied. Another important condition is that the interval of acaricide treatment may have a strong correlation to tick infestation and differences in milk quality and production but these data still need to be definite.

## Conclusions

We have confirmed that the indiscriminate use of acaricides can be one of the main causes of tick resistance, as described by the individual farm management. The AIT, the EHT, and the OE showed a similar trend in determining efficacy for the best compound (G4, G3, G2, and G1). G4 and G3 showed 100% efficacy in the PRA farm after a long withhold period of 15 years. It was also possible to conclude that G1 and G3 that had drug combinations did not represent a chemical advantage, even though they could be used once their individual efficacy is previously determined.
